# Crystal structure of BaMnB_2_O_5_ containing structurally isolated manganese oxide sheets

**DOI:** 10.1107/S2056989016013074

**Published:** 2016-08-19

**Authors:** Elizabeth M. Maschmeyer, Liurukara D. Sanjeewa, Kulugammana G. S. Ranmohotti

**Affiliations:** a Division of Chemistry and Biological Sciences, Governors State University, 1 University Parkway, University Park, IL 60484-0975, USA; b Department of Chemistry, Clemson University, Clemson, SC 29634-0973, USA

**Keywords:** crystal structure, pyroborates, inter­planar angle, layered framework, bond-valence-sum calculations

## Abstract

In an attempt to search for mixed alkaline-earth and transition metal pyroborates, the title compound, barium manganese(II) pyroborate, has been synthesized by employing a flux method. The structure of BaMnB_2_O_5_ is composed of MnO_5_ square pyramids that form Mn_2_O_8_ dimers by edge-sharing and of pyroborate units ([B_2_O_5_]^4−^) that are composed of two corner-sharing trigonal–planar BO_3_ units. These building blocks share corners to form _∞_^2^[MnB_2_O_5_]^2−^ layers extending parallel to (100). The Ba^2+^ cations reside in the gap between two manganese pyroborate slabs with a coordination number of nine. The title compound forms an inter­esting spiral framework propagating along the 2_1_ screw axis. The structure is characterized by two alternating layers, which is relatively rare among known transition-metal-based pyroborate compounds.

## Abstract 2

The structure of BaMnB_2_O_5_ is characterized by infinite sheets of B_2_O_5_ units and Mn_2_O_8_ dimers of edge-sharing MnO_5_ square pyramids while Ba^2+^ cations inter­leave successive sheets.

## Chemical context   

Numerous borates with various crystal structures and compositions have been widely investigated over the last few decades (Heller *et al.*, 1986 ▸). Pyroborates containing the (B_2_O_5_)^4−^ anion were first structurally characterized in 1950 (Berger, 1950 ▸). Pyroborates can be divided into two subclasses such as alkaline-earth-based pyroborates with general formula *A*_2_B_2_O_5_ (*A* = alkaline earth metal) and transition-metal-based pyroborates with general formula *MM*′B_2_O_5_. If *M* = *M*′, the pyroborate is considered to be homo-metallic, otherwise it is hetero-metallic.

Alkaline-earth-based pyroborates adopt different structure types. During the investigation of the BaO/B_2_O_3_ system, Hubner revealed Ba_2_B_2_O_5_ crystallizing in space group *P*2/*m* (Hubner, 1969 ▸). The other alkaline-earth-based *A*_2_B_2_O_5_ pyroborates (*A* = Mg, Ca, Sr) have been synthesized by high-temperature solid-state reactions. Mg_2_B_2_O_5_ (Guo *et al.*, 1995*b* ▸) crystallizes in space group *P*2_1_ */c*. Ca_2_B_2_O_5_ (Lin *et al.*, 1999*a* ▸) and Sr_2_B_2_O_5_ (Lin *et al.*, 1999*b* ▸) are isotypic and crystallize in the same space group type as Mg_2_B_2_O_5_ but have a different structure from the latter. Additionally, there exists a triclinic magnesium pyroborate (*P* 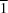; Guo *et al.*, 1995*a* ▸). The existence of mixed alkaline-earth-based pyroborates (*AA*′B_2_O_5_) has been proven by the study of naturally occurring minerals. The crystal structures of two polymorphs of CaMgB_2_O_5_, kurchatovite and clinokurchatovite, have been originally determined in space group types *Pc*2_1_ *b* (Yakubovich *et al.*, 1976 ▸) and *P*2_1_/*c* (Simonov *et al.*, 1980 ▸). However, the crystal structures of both minerals have been re-examined and refined in different space group types (Callegari *et al.*, 2003 ▸). Based on these models, kurchatovite crystallizes in space group type *Pbca* whilst clinokurchatovite crystallizes in space group type *P*2_1_/*c*.

Investigations of transition-metal-based homo-metallic pyroborates, *M*_2_B_2_O_5_ have led to four compounds, namely Mn_2_B_2_O_5_ (Sarrat *et al.*, 2005 ▸), Co_2_B_2_O_5_ (Rowsell *et al.*, 2003 ▸), Cd_2_B_2_O_5_ (Weil, 2003 ▸), and Fe_2_B_2_O_5_ (Neumair & Huppertz, 2009 ▸). These phases crystallize isotypically with the triclinic form of Mg_2_B_2_O_5_ (Guo *et al.*, 1995*a* ▸). Efforts have been made to isolate transition-metal-based hetero-metallic phases, *MM*′B_2_O_5_. This has resulted in the synthesis of MnCoB_2_O_5_ and MnMgB_2_O_5_ (Utzolino & Bluhm, 1996 ▸), and Ni_1.5_Zn_0.5_B_2_O_5_ and Co_1.5_Zn_0.5_B_2_O_5_ (Busche & Bluhm, 1995 ▸). These structures are also isotypic with the triclinic form of Mg_2_B_2_O_5_.

Investigations of the BaO/CuO/B_2_O_3_ phase diagram has resulted in the isolation of a non-centrosymmetric pyroborate, BaCuB_2_O_5_ (Smith & Keszler, 1997 ▸) with a unique structure type in space group type *C*2. As part of an effort to isolate new mixed alkaline earth and transition metal pyroborates, we have investigated the BaO/MnO/B_2_O_3_ phase diagram. In this study, we have grown single crystals of BaMnB_2_O_5_ and analyzed its crystal structure.

## Structural commentary   

**Figure 1:**
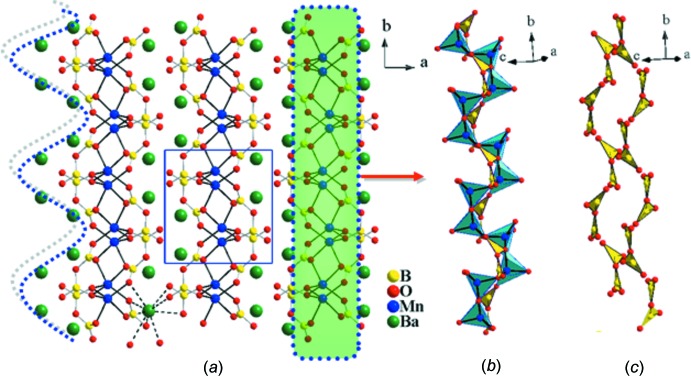
(*a*) Perspective view of the structure of BaMnB_2_O_5_ viewed along the *c* axis. The wavy and dotted line (left) indicates the zigzag arrays of Ba atoms. Only one Ba atom with bonds is drawn for clarity, demonstrating the function of Ba—O bonds with regard to holding neighboring [MnB_2_O_5_]^2−^ slabs. (*b*) Polyhedral representation showing the ^2^_∞_[MnB_2_O_5_]^2−^ spiral framework centered around the 2_1_ screw axis of the unit cell. (*c*) Polyhedral representation showing the arrangement of isolated pyroborate units viewed approximately along the [101] direction.

**Figure 2:**
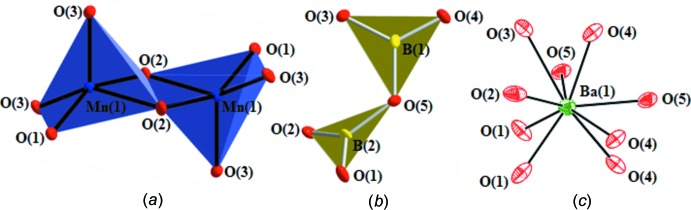
(*a*) Partial structure showing Mn_2_O_8_ dimers (polyhedral drawing). The apical oxygen, O3, in each MnO_5_ square pyramid points in opposite directions. (*b*) Corner-sharing BO_3_ groups forming a pyroborate unit. The BO_3_ units within the pyroborate group are linked through a common O atom, O5. (*c*) The barium cation resides in a BaO_9_ environment. Anisotropic displacement parameters are drawn at 95% probability.

The crystal structure of BaMnB_2_O_5_ defines a new structure type that can be described as being composed of manganese pyroborate slabs with composition _∞_^2^[MnB_2_O_5_]^2−^ that extend parallel to (100). Fig. 1 ▸ *a* shows a perspective drawing of the BaMnB_2_O_5_ structure with the quasi-two-dimensional lattice characterized by [MnB_2_O_5_]^2−^ slabs. The barium cations reside between the parallel slabs and maintain the inter­slab connectivity through coordination to nine oxygen atoms (Fig. 2 ▸ *c*).

Two non-equivalent boron atoms are present in the structure; both are surrounded by three oxygen atoms to form almost regular trigonal–planar units. As depicted in Fig. 2 ▸ *b*, the isolated [B_2_O_5_]^4−^ pyroborate groups are composed of two corner-sharing trigonal–planar BO_3_ units. In the reported pyroborate structures (Thompson *et al.*, 1991 ▸), the terminal BO_2_ planes pivot about the torsion angles to afford deviations from coplanarity that can range from 0 to 76.8° where the B—O—B angle ranges from 112 to 180°. In BaMnB_2_O_5_, the pyroborate groups show closely related geometric features as previously noted (Thompson *et al.*, 1991 ▸), exhibiting a B—O—B angle of 125.1 (5)° whilst the dihedral angle between the two BO_3_ units within the pyroborate group is 48.62 (8)°. The asymmetry of the bond lengths in the B_2_O_5_ group is indicated by slightly varied bond lengths of terminal and bridging B—O bonds. The bridging B—O bond lengths are slightly longer [B1—O5: 1.423 (7); B2—O5: 1.432 (7) Å] than the terminal B—O bond lengths [range 1.332 (8)–1.384 (7) Å]. Notwithstanding, the average B—O bond length (1.380 Å) in the title compound is very close to the corresponding average B—O bond length in BO_3_ groups in previously reported borates (1.370 Å; Zobetz, 1982 ▸). Fig. 1 ▸ *c* shows the arrangement of isolated pyroborate units, appearing as two parallel pseudo-one-dimensional chains spiraling around the 2_1_ axis.

**Figure 3:**
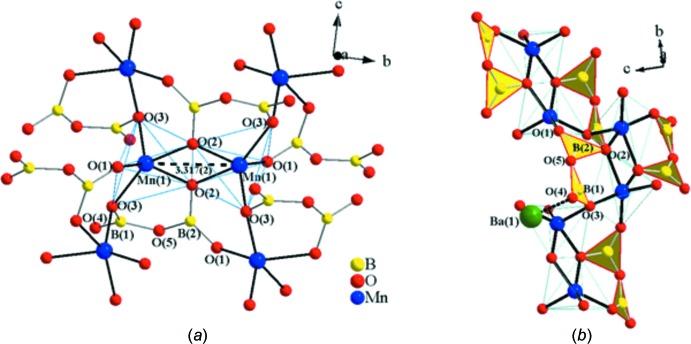
(*a*) The Mn1O_5_ square pyramids (ball and stick drawing) share a common edge, O2—O2, forming an Mn_2_O_8_ unit. (*b*) The B2O_3_ unit (polyhedral drawing), shares two corners with neighboring MnO_5_ square pyramids (ball and stick drawing) through O1 and O2. The only unshared oxygen, O4, of the pyroborate group forms a bond with a Ba atom.

There is one crystallographically independent Mn atom which is coordinated by five oxygen atoms to form a square pyramid with four longer equatorial Mn—O bonds and one short apical Mn—O bond. Fig. 2 ▸ *a* shows two Mn1O_5_ square pyramids sharing a common edge, O2—O2(−*x* + 1, −*y*, −*z*), to form an Mn_2_O_8_ unit. As shown in Fig. 3 ▸ *a*, Mn atoms are connected to each other *via* oxygen atoms with a Mn1⋯Mn1 separation of 3.317 (2) Å and an Mn1—O2—Mn1 angle of 101.23 (16)°. The neighboring Mn_2_O_8_ dimers share vertices through oxygen atom O3. The oxygen atom O1 in the Mn_2_O_8_ dimer is only corner-shared by the pyroborate group. The only unshared oxygen, O4, of the pyroborate group is pointing into the free space towards the neighboring slabs to form a bond with the barium atom. As shown in Fig. 3 ▸ *b*, with respect to the pyroborate group, the B2O_3_ unit shares two corners with neighboring MnO_5_ square pyramids through O1 and O2 while the B1O_3_ unit corner-shares a common oxygen atom, O3, with two other MnO_5_ square pyramids. This arrangement facilitates the observed curvature which is necessary for the spiral framework found in the extended lattice (Fig. 1 ▸ *b*). The unique arrangement of B_2_O_5_ groups around the 2_1_ screw axis provides an essential element allowing the spiral chain to propagate along the *b* axis. It is well known that the inter­planar angle of the B_2_O_5_ group is primarily dictated by packing effects and the nature of the associated cations in the given structure (Thompson *et al.*, 1991 ▸). In addition to that, as previously noted, the greater deviations from coplanarity are observed in the arrangement of the B_2_O_5_ groups due to variation of the sizes of alkali metals in alkali metal Nb and Ta oxide pyroborates (Akella & Keszler, 1995 ▸). Accordingly, the inter-planar angle of the B_2_O_5_ group is likely to be determined by the associate coordination environment of the barium cations in the title compound. It should be noted that the connectivity of the Mn_2_O_8_ and B_2_O_5_ structure units would result in a ‘dangling’ framework unless it can be tightly held together by external bonds. The Ba^2+^ cations, in this case, reside in the spiral framework arranging in zigzag fashion to support and maintain the distance between neighboring [MnB_2_O_5_]^2−^ slabs. Coincidentally, this wavy arrangement is critical for the spiral chain to propagate along the *b* axis. The flexible [MnB_2_O_5_]^2−^ framework revolves around Ba^2+^ cations, suggesting a template-like behavior.

The MnO_5_ units adopt bond lengths normally observed in the Mn^II^ borates. The Mn^2+^—O bond lengths range from 2.082 (4) to 2.151 (4) Å, comparable with 2.10 Å, the sum of the Shannon crystal radii (Shannon, 1976 ▸) for a five coordin­ated Mn^2+^ (0.89 Å) and two-coordinated O^2−^ (1.21 Å). The bond-valence-sum (BVS) calculations (Brese & O’Keeffe, 1991 ▸) for BaMnB_2_O_5_ give a valence unit (v.u.) of 2.03 for the Mn^2+^ cation. Based on parameters for B^3+^—O, bond-valence sums of 2.95 for B1^3+^ and 2.95 for B2^3+^ were calculated. The Ba—O bond lengths are quite diverse, ranging from 2.707 (4) to 2.958 (4) Å. The average Ba—O bond length, 2.83 Å, however matches closely with 2.82 Å, the sum of the Shannon crystal radii for a nine coordinated Ba^2+^ (1.61 Å) and two-coordinated O^2−^ (1.21 Å). The BVS calculation for Ba^2+^ results in 2.08 v.u.

**Figure 4:**
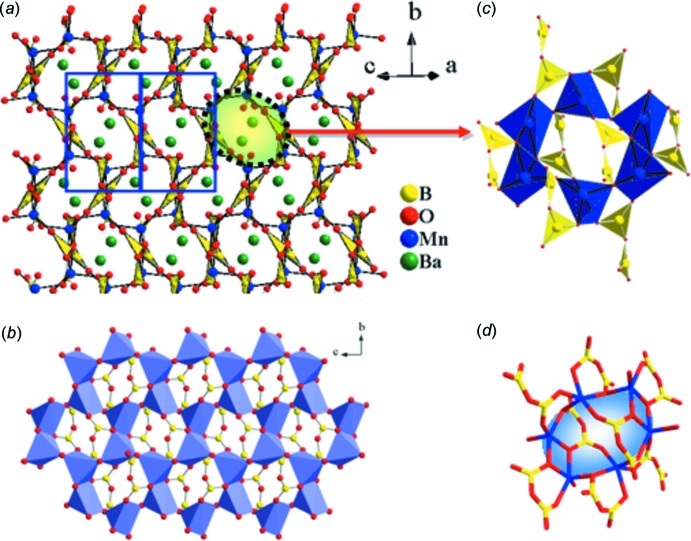
(*a*) Extended structure of BaMnB_2_O_5_ viewed approximately along the [101] direction. The connectivity of the barium atoms is not shown for clarity. (*b*) Partial structure of [MnB_2_O_5_]^2−^ slab viewed along [100] where the polyhedral drawing represents MnO_5_ square pyramids and B_2_O_5_ units are represented by ball-and-stick drawing. (*c*) Edge-sharing and corner-sharing MnO_5_ units corner-share with B_2_O_5_ pyroborate groups (polyhedral drawing) to create a cage. (*d*) Stick drawing of one cage with empty space in the middle.

**Figure 5:**
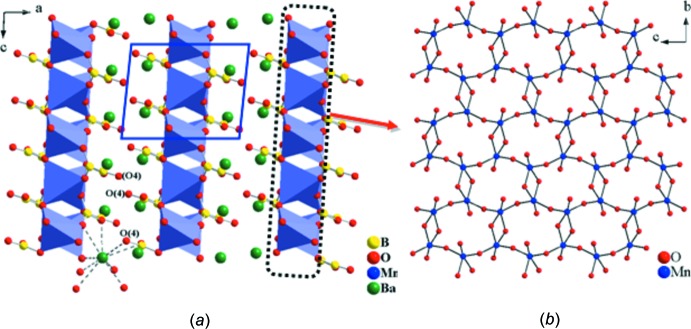
(*a*) Layered BaMnB_2_O_5_ shown by polyhedral and ball-and-stick drawing viewed along the [010] direction. (*b*) Ball-and-stick drawing of a portion of the manganese oxide network formed by inter­connected Mn_2_O_8_ dimers by corner sharing.

One of the inter­esting features of the title compound is that the structure can be alternatively viewed as a ‘porous’ framework as shown in Fig. 4 ▸ *a*. The B_2_O_5_ units together with inter­connected Mn_2_O_8_ dimers extend along the *b* axis in a standing wave fashion, creating oval shape windows which also arrange in a zigzag fashion along the same direction. It is intriguing to notice that the two B_2_O_5_ groups along with the two Mn_2_O_8_ dimers and two MnO_5_ square pyramids create an empty cage (Fig. 4 ▸ *c,d*). The polyhedral and ball-and-stick drawing (Fig. 4 ▸ *b*) clearly shows the three-dimensional framework bearing large cavities. This unusual structural arrangement is conceivably attributed to the limitation of the size of the pyroborate unit that simultaneously tends to inter­connect with barium cations and neighboring Mn_2_O_8_ dimers in a corner-shared fashion (Fig. 3 ▸ *b*). As shown in Fig. 5 ▸ *a*, the layered nature of the title compound is characterized by parallel [MnB_2_O_5_]^2−^ slabs outlined by a dotted rectangle viewed along [010]. Fig. 5 ▸ *b* shows the ball-and-stick drawing of a portion of the layered manganese oxide network. Each Mn_2_O_8_ dimer shares vertices with four other MnO_5_ square pyramids through oxygen atoms to form these sheets. Within the extended Mn—O sheet, the MnO_5_ square pyramids which share edges are separated from each other by 3.317 (2) Å (Mn1⋯Mn1 distance) whereas those which share corners are separated by 3.435 (1) Å. The distance between the Mn atoms of the adjacent sheets is 8.287 (2) Å. Given the description of the local configuration of the manganese oxide polyhedra, their connectivity along the sheet, and the structural isolation of neighboring Mn—O sheets from each other, one would suspect that the title compound offers opportunities for the study of spin exchange in a confined Mn—O lattice. In theory, a periodic array of well-defined transition metal oxide lattices could provide a useful model for experimental and theoretical developments of magnetic and electronic inter­actions in transition metal oxides because of their simplified structures (Snyder *et al.*, 2001 ▸).

The structure of BaMnB_2_O_5_ is somewhat related to that of triclinic *M*_2_B_2_O_5_ (*M* = Mn, Sarrat *et al.*, 2005 ▸; Fe, Neumair & Huppertz, 2009 ▸; Co, Rowsell *et al.*, 2003 ▸) phases. The structures of *M*_2_B_2_O_5_ (*M* = Mn, Fe, Co) phases are based on structurally isolated [*M*_4_O_18_] tetra­mers, composed of four octa­hedra linked by three shared edges, connected through sharing four O—O edges, into ribbons extending along (001) while the boron atoms hold the ribbons together forming B_2_O_5_ groups. These extended ribbons are parallel to each other and therefore these *M*_2_B_2_O_5_ phases have a quasi-one-dimensional structure in contrast to the two-dimensional Mn—O lattice in the title compound. Magnetic properties have been widely studied for Co_2_B_2_O_5_ (Kawano *et al.*, 2010 ▸), Fe_2_B_2_O_5_ (Kawano *et al.*, 2009 ▸), and Mn_2_B_2_O_5_ (Fernandes *et al.*, 2003 ▸) to understand the low-dimensional inter­actions derived from the ribbon-like substructures in these compounds. The spin configuration based on the electron-density distribution has been proposed (Sarrat *et al.*, 2005 ▸) for Mn_2_B_2_O_5_ in which the distance between manganese atoms of adjacent ribbons are 4.526–6.272 Å and electron-density distributions were indicated in the regions between the ribbons. According to their model, all coplanar ribbons of Mn_2_B_2_O_5_ are ferromagnetic; their anti­ferromagnetic behavior is derived from anti­parallel magnetic orientations between adjacent ribbons. In the title compound, the distance between manganese atoms within the sheets and adjacent sheets are 3.317 (2)–3.435 (1) Å and 8.287 (2) Å, respectively. It is important to note that the Ba^2+^ cations reside in the gap between the two Mn—O sheets. This, together with the greater separation between manganese atoms of adjacent sheets, leads us to believe that magnetic inter­actions that occur between Mn—O sheets can be extremely weak and the dominant magnetic exchange should be between Mn^2+^ ions within the Mn—O sheet. Judging from the inter­esting magnetic properties reported for *M*_2_B_2_O_5_ (*M* = Mn, Fe, Co) compounds, we expect inter­esting magnetic phenomena from a systematic investigation of the magnetic susceptibility of BaMnB_2_O_5_.

## Synthesis and crystallization   

Light pink crystals of BaMnB_2_O_5_ were grown by employing a CsCl/RbCl flux in a fused silica ampoule under vacuum. MnO (2.74 mmol, 99.999+%, Alfa), BaO (1.37 mmol, 99.99+%, Aldrich) and B_2_O_3_ (1.37 mmol, 99.98+%, Aldrich) were mixed and ground with the flux (1:3 by weight) in a nitro­gen-blanketed drybox. The resulting mixture was heated to 818 K at 1 K min^−1^, isothermed for two days, heated to 1023 K at 1 K min^−1^, isothermed for four days, then slowly cooled to 673 K at 0.1 K min^−1^ followed by furnace-cooling to room temperature. Prismatic crystals of BaMnB_2_O_5_ were retrieved upon washing off the solidified melt with deionized water.

## Refinement   

Crystal data, data collection and structure refinement details are summarized in Table 1 ▸. The final Fourier difference synthesis showed the maximum residual electron density in the difference Fourier map, 0.82 e Å^−3^, located at 1.19 Å from Ba1 and the minimum, −0.98 e^−^ Å^−3^, at 0.92 Å from Ba1.

## Supplementary Material

Crystal structure: contains datablock(s) I. DOI: 10.1107/S2056989016013074/wm5306sup1.cif

Structure factors: contains datablock(s) I. DOI: 10.1107/S2056989016013074/wm5306Isup2.hkl

CCDC reference: 1499124

Additional supporting information: crystallographic information; 3D view; checkCIF report

## Figures and Tables

**Table 1:** Experimental details

Crystal data	
Chemical formula	BaMnB_2_O_5_
*M*_r_	293.90
Crystal system, space group	Monoclinic, *P*2_1_/*c*
Temperature (K)	300
*a*, *b*, *c* (Å)	8.2868 (17), 8.6570 (17), 6.5263 (13)
β (°)	92.87 (3)
*V* (Å^3^)	467.60 (16)
*Z*	4
Radiation type	Mo *K*α
μ (mm^−1^)	10.99
Crystal size (mm)	0.17 × 0.05 × 0.02

Data collection	
Diffractometer	Rigaku AFC8S
Absorption correction	Multi-scan (*REQAB*; Rigaku, 1998 ▸)
*T*_min_, *T*_max_	0.518, 0.808
No. of measured, independent and observed [*I* > 2σ(*I*)] reflections	3807, 830, 777
*R*_int_	0.052
(sin θ/λ)_max_ (Å^−1^)	0.598

Refinement	
*R*[*F* ^2^ > 2σ(*F* ^2^)], *wR*(*F* ^2^), *S*	0.025, 0.056, 1.10
No. of reflections	830
No. of parameters	83
Δρ_max_, Δρ_min_ (e Å^−3^)	0.82, −0.98

Computer programs: *CrystalClear* (Rigaku, 2006 ▸), *SHELXT* (Sheldrick, 2015*a* ▸), *SHELXL2014* (Sheldrick, 2015*b* ▸), *DIAMOND* (Brandenburg, 1999 ▸) and *publCIF* (Westrip, 2010 ▸).

## supplementary crystallographic information

### Crystal data

**Table:**

BaMnB_2_O_5_	*F*(000) = 524
*M**_r_* = 293.90	*D*_x_ = 4.175 Mg m^−^^3^
Monoclinic, *P*2_1_/*c*	Mo *K*α radiation, λ = 0.71073 Å
*a* = 8.2868 (17) Å	Cell parameters from 3139 reflections
*b* = 8.6570 (17) Å	θ = 3.1–29.6°
*c* = 6.5263 (13) Å	µ = 10.99 mm^−^^1^
β = 92.87 (3)°	*T* = 300 K
*V* = 467.60 (16) Å^3^	Prismatic, pink
*Z* = 4	0.17 × 0.05 × 0.02 mm

### Data collection

**Table:**

Rigaku AFC8S diffractometer	777 reflections with *I* > 2σ(*I*)
Radiation source: fine-focus sealed tube	*R*_int_ = 0.052
ω scans	θ_max_ = 25.2°, θ_min_ = 3.4°
Absorption correction: multi-scan (*REQAB*; Rigaku, 1998)	*h* = −9→9
*T*_min_ = 0.518, *T*_max_ = 0.808	*k* = −10→10
3807 measured reflections	*l* = −7→7
830 independent reflections	1 standard reflections every 1 reflections

### Refinement

**Table:**

Refinement on *F*^2^	0 restraints
Least-squares matrix: full	*w* = 1/[σ^2^(*F*_o_^2^) + (0.0315*P*)^2^] where *P* = (*F*_o_^2^ + 2*F*_c_^2^)/3
*R*[*F*^2^ > 2σ(*F*^2^)] = 0.025	(Δ/σ)_max_ = 0.005
*wR*(*F*^2^) = 0.056	Δρ_max_ = 0.82 e Å^−^^3^
*S* = 1.10	Δρ_min_ = −0.98 e Å^−^^3^
830 reflections	Extinction correction: SHELXL2014 (Sheldrick, 2015b), Fc^*^=kFc[1+0.001xFc^2^λ^3^/sin(2θ)]^-1/4^
83 parameters	Extinction coefficient: 0.0061 (7)

### Special details

**Table:**

Geometry. All esds (except the esd in the dihedral angle between two l.s. planes) are estimated using the full covariance matrix. The cell esds are taken into account individually in the estimation of esds in distances, angles and torsion angles; correlations between esds in cell parameters are only used when they are defined by crystal symmetry. An approximate (isotropic) treatment of cell esds is used for estimating esds involving l.s. planes.

### Fractional atomic coordinates and isotropic or equivalent isotropic displacement parameters (Å^2^)

**Table:**

	*x*	*y*	*z*	*U*_iso_*/*U*_eq_	
Ba1	0.14891 (4)	0.07969 (4)	0.22488 (5)	0.00932 (16)	
Mn1	0.53148 (10)	0.18805 (10)	0.02929 (12)	0.0087 (2)	
B1	0.1784 (8)	−0.2828 (7)	0.1447 (10)	0.0100 (13)	
B2	0.2960 (8)	0.4492 (7)	0.1980 (10)	0.0115 (13)	
O1	0.3314 (5)	0.3308 (4)	0.0723 (6)	0.0133 (9)	
O2	0.3587 (5)	0.0300 (4)	−0.1080 (6)	0.0126 (9)	
O3	0.3228 (5)	−0.2185 (4)	0.2184 (6)	0.0106 (8)	
O4	0.0457 (5)	−0.2018 (4)	0.0939 (6)	0.0110 (8)	
O5	−0.1725 (5)	0.0536 (4)	0.3763 (6)	0.0109 (8)	

### Atomic displacement parameters (Å^2^)

**Table:**

	*U*^11^	*U*^22^	*U*^33^	*U*^12^	*U*^13^	*U*^23^
Ba1	0.0103 (2)	0.0088 (2)	0.0088 (2)	0.00002 (12)	−0.00031 (13)	−0.00026 (13)
Mn1	0.0094 (5)	0.0080 (4)	0.0086 (5)	0.0000 (3)	−0.0006 (3)	−0.0001 (3)
B1	0.006 (3)	0.013 (3)	0.011 (3)	−0.002 (3)	0.000 (2)	0.002 (3)
B2	0.010 (3)	0.011 (3)	0.014 (3)	0.005 (3)	0.000 (3)	−0.003 (3)
O1	0.012 (2)	0.015 (2)	0.013 (2)	0.0056 (17)	−0.0030 (17)	−0.0054 (17)
O2	0.017 (2)	0.0073 (18)	0.012 (2)	−0.0008 (16)	−0.0055 (17)	−0.0004 (16)
O3	0.0108 (19)	0.0109 (19)	0.010 (2)	−0.0023 (16)	−0.0002 (16)	0.0011 (16)
O4	0.012 (2)	0.009 (2)	0.012 (2)	0.0033 (16)	0.0004 (16)	−0.0018 (16)
O5	0.013 (2)	0.0075 (19)	0.012 (2)	0.0010 (16)	−0.0036 (16)	0.0006 (16)

### Geometric parameters (Å, º)

**Table:**

Ba1—O4	2.707 (4)	Mn1—O1	2.098 (4)
Ba1—O1^i^	2.772 (4)	Mn1—O2	2.144 (4)
Ba1—O4^ii^	2.777 (4)	Mn1—O2^v^	2.147 (4)
Ba1—O4^iii^	2.787 (4)	Mn1—O3^vi^	2.151 (4)
Ba1—O5^iv^	2.845 (4)	B1—O4	1.332 (8)
Ba1—O1	2.855 (4)	B1—O3	1.384 (7)
Ba1—O2	2.882 (4)	B1—O5^vii^	1.423 (7)
Ba1—O5	2.896 (4)	B2—O1	1.354 (7)
Ba1—O3	2.958 (4)	B2—O2^i^	1.357 (8)
Mn1—O3^v^	2.082 (4)	B2—O5^iii^	1.432 (7)
			
O4—Ba1—O1^i^	131.36 (11)	O1—Mn1—O2^v^	144.52 (16)
O4—Ba1—O4^ii^	86.77 (11)	O2—Mn1—O2^v^	78.77 (16)
O1^i^—Ba1—O4^ii^	141.16 (11)	O3^v^—Mn1—O3^vi^	102.81 (15)
O4—Ba1—O4^iii^	124.37 (5)	O1—Mn1—O3^vi^	95.36 (15)
O1^i^—Ba1—O4^iii^	76.50 (12)	O2—Mn1—O3^vi^	153.85 (15)
O4^ii^—Ba1—O4^iii^	74.51 (8)	O2^v^—Mn1—O3^vi^	86.12 (14)
O4—Ba1—O5^iv^	85.98 (11)	O1—B2—O2^i^	125.6 (5)
O1^i^—Ba1—O5^iv^	49.87 (11)	O1—B2—O5^iii^	116.5 (5)
O4^ii^—Ba1—O5^iv^	148.29 (11)	O2^i^—B2—O5^iii^	117.8 (5)
O4^iii^—Ba1—O5^iv^	84.37 (11)	B2—O1—Mn1	136.3 (4)
O4—Ba1—O1	137.70 (12)	B2—O1—Ba1^viii^	99.1 (3)
O1^i^—Ba1—O1	78.21 (9)	Mn1—O1—Ba1^viii^	117.24 (16)
O4^ii^—Ba1—O1	75.33 (12)	B2—O1—Ba1	103.3 (3)
O4^iii^—Ba1—O1	87.65 (11)	Mn1—O1—Ba1	91.97 (13)
O5^iv^—Ba1—O1	127.91 (11)	Ba1^viii^—O1—Ba1	102.80 (13)
O4—Ba1—O2	79.67 (11)	B2^viii^—O2—Mn1	121.5 (4)
O1^i^—Ba1—O2	109.48 (11)	B2^viii^—O2—Mn1^v^	118.6 (4)
O4^ii^—Ba1—O2	80.88 (11)	Mn1—O2—Mn1^v^	101.23 (16)
O4^iii^—Ba1—O2	143.59 (11)	B2^viii^—O2—Ba1	117.8 (4)
O5^iv^—Ba1—O2	127.79 (11)	Mn1—O2—Ba1	90.29 (13)
O1—Ba1—O2	60.05 (11)	Mn1^v^—O2—Ba1	102.44 (14)
O4—Ba1—O5	75.88 (11)	B1—O3—Mn1^v^	107.8 (4)
O1^i^—Ba1—O5	102.48 (11)	B1—O3—Mn1^ix^	123.9 (3)
O4^ii^—Ba1—O5	77.04 (12)	Mn1^v^—O3—Mn1^ix^	108.48 (17)
O4^iii^—Ba1—O5	49.20 (11)	B1—O3—Ba1	86.7 (3)
O5^iv^—Ba1—O5	71.25 (13)	Mn1^v^—O3—Ba1	101.68 (14)
O1—Ba1—O5	133.67 (10)	Mn1^ix^—O3—Ba1	124.92 (15)
O2—Ba1—O5	147.72 (11)	B1—O4—Ba1	98.7 (3)
O4—Ba1—O3	49.93 (11)	B1—O4—Ba1^ii^	145.9 (4)
O1^i^—Ba1—O3	90.55 (11)	Ba1—O4—Ba1^ii^	93.23 (11)
O4^ii^—Ba1—O3	125.91 (11)	B1—O4—Ba1^vii^	91.4 (3)
O4^iii^—Ba1—O3	153.01 (10)	Ba1—O4—Ba1^vii^	131.11 (15)
O5^iv^—Ba1—O3	69.38 (11)	Ba1^ii^—O4—Ba1^vii^	104.46 (13)
O1—Ba1—O3	113.11 (11)	B1^iii^—O5—B2^vii^	125.1 (5)
O2—Ba1—O3	63.01 (10)	B1^iii^—O5—Ba1^iv^	119.5 (3)
O5—Ba1—O3	113.20 (11)	B2^vii^—O5—Ba1^iv^	93.9 (3)
O3^v^—Mn1—O1	121.48 (15)	B1^iii^—O5—Ba1	85.3 (3)
O3^v^—Mn1—O2	99.07 (15)	B2^vii^—O5—Ba1	126.0 (3)
O1—Mn1—O2	85.17 (16)	Ba1^iv^—O5—Ba1	108.75 (13)
O3^v^—Mn1—O2^v^	92.39 (15)		

Symmetry codes: (i) *x*, −*y*+1/2, *z*+1/2; (ii) −*x*, −*y*, −*z*; (iii) −*x*, *y*+1/2, −*z*+1/2; (iv) −*x*, −*y*, −*z*+1; (v) −*x*+1, −*y*, −*z*; (vi) −*x*+1, *y*+1/2, −*z*+1/2; (vii) −*x*, *y*−1/2, −*z*+1/2; (viii) *x*, −*y*+1/2, *z*−1/2; (ix) −*x*+1, *y*−1/2, −*z*+1/2.
